# Statistical Inter-stimulus Interval Window Estimation for Transient Neuromodulation via Paired Mechanical and Brain Stimulation

**DOI:** 10.3389/fnbot.2020.00001

**Published:** 2020-02-03

**Authors:** Euisun Kim, Waiman Meinhold, Minoru Shinohara, Jun Ueda

**Affiliations:** ^1^Bio-Robotics and Human Modeling Laboratory, G.W.W. School of Mechanical Engineering, Georgia Institute of Technology, Atlanta, GA, United States; ^2^Human Neuromuscular Physiology Laboratory, School of Biological Sciences, Georgia Institute of Technology, Atlanta, GA, United States

**Keywords:** brain stimulation, transcranial magnetic stimulation, mechanical stimulation, motor evoked potential, statistical estimation

## Abstract

For achieving motor recovery in individuals with sensorimotor deficits, augmented activation of the appropriate sensorimotor system, and facilitated induction of neural plasticity are essential. An emerging procedure that combines peripheral nerve stimulation and its associative stimulation with central brain stimulation is known to enhance the excitability of the motor cortex. In order to effectively apply this paired stimulation technique, timing between central and peripheral stimuli must be individually adjusted. There is a small range of effective timings between two stimuli, or the inter-stimulus interval window (ISI-W). Properties of ISI-W from neuromodulation in response to mechanical stimulation (Mstim) of muscles have been understudied because of the absence of a versatile and reliable mechanical stimulator. This paper adopted a combination of transcranial magnetic stimulation (TMS) and Mstim by using a high-precision robotic mechanical stimulator. A pneumatically operated robotic tendon tapping device was applied. A low-friction linear cylinder achieved high stimulation precision in time and low electromagnetic artifacts in physiological measurements. This paper describes a procedure to effectively estimate an individual ISI-W from the transiently enhanced motor evoked potential (MEP) with a reduced number of paired Mstim and sub-threshold TMS trials by applying statistical sampling and regression technique. This paper applied a total of four parametric and non-parametric statistical regression methods for ISI-W estimation. The developed procedure helps to reduce time for individually adjusting effective ISI, reducing physical burden on the subject.

## 1. Introduction

Upper limb motor function is commonly degraded with aging and is often impaired due to neurologic injuries such as stroke and spinal cord injury. Of the 658,000 stroke survivors in the U.S. annually, approximately 60% of stroke survivors experience significant impairments in hand function, requiring long-term rehabilitative therapy to regain function (Dam et al., 1993). There are few interventions that improve upper limb function. A lack of experimental tools and procedures has been a barrier in studying neuromodulation in response to peripheral mechanical stimulation that is analogous to physical therapy for individuals with hemiparesis.

### 1.1. Paired Associative Stimulation for Neural Facilitation

Neural excitability of the motor cortex is essential for activating muscles, but is often compromised in hemiparetic stroke survivors, while modulation of this excitability can facilitate motor learning and recovery (Hummel and Cohen, 2006; Kim et al., 2006; Jung and Ziemann, 2009; Lazzaro et al., 2009; Teo et al., 2010). Excitability of the motor cortex is modulated by a repetition of electrical stimulation (Estim) to a peripheral nerve (e.g., median nerve) alone or paired with central brain stimulation known as Paired Associative Stimulation (PAS) (Stefan, 2000; Wolters et al., 2003; Meunier et al., 2007; Kennedy and Carson, 2008; Carson and Kennedy, 2013). Excitability of the primary motor cortex (M1) can be potentiated in the long term (approximately 1 h) after a repetition of a conventional form of PAS, i.e., application of Estim to the peripheral nerve (e.g., median nerve) with an appropriate time interval immediately (approximately 25 ms) before transcranial magnetic stimulation (TMS) to M1 (termed Estim-PAS in this paper) in experimental studies (Stefan, 2000; Wolters et al., 2003; Meunier et al., 2007; Kennedy and Carson, 2008; Carson and Kennedy, 2013).

In a therapeutic analogy, as an emerging effective rehabilitation for hemiparetic stroke survivors, a repetition of a manual application of mechanical stimulation (Mstim) to the target muscles (e.g., tendon tapping or rapid muscle stretch) by a therapist immediately before a contraction effort of a patient (termed Mstim-paired manual therapy in this paper) can facilitate neuromotor recovery and often lead to significantly better rehabilitation outcomes compared with the conventional rehabilitation without Mstim (Kawahira et al., 2004, 2009, 2010; Shimodozono et al., 2012; Kawakami et al., 2015; Matsumoto et al., 2016).

One of the difficulties of stroke rehabilitation is that neurorehabilitation efficacy is variable (Langhorne et al., 2011; Pollock et al., 2014; Veerbeek et al., 2014). Since a repeated application of PAS can result in facilitation or inhibition of neural excitability depending on the timing interval of stimulations, determination of the appropriate inter-stimulus interval (ISI) is crucial for inducing facilitation (Poon et al., 2008; Kumpulainen et al., 2012). ISI is the period of time that separates two consecutive stimuli (Pereira et al., 2014). In the conventional form of PAS (i.e., Estim-PAS), ISI is the timing difference between Estim and TMS. Neural plasticity via Hebbian learning is assumed to be induced through the repetition of paired associative excitatory inputs to M1, one originating from the stimulation of the somatosensory system (afferents) and another from TMS or motor effort of an individual in response to therapist's instruction (Takeuchi and Izumi, 2015) that arrives at M1 shortly after the peripheral stimulation. The time interval between two paired inputs is critical to the consequence of PAS and likely other therapeutic techniques (Wolters et al., 2005; Takeuchi and Izumi, 2015). Since the scientific mechanisms for this promising emerging Mstim-paired manual therapy are not well understood, the time interval between the peripheral Mstim by a therapist and central motor effort by a patient is undefined and thus can be variable. Individual differences in signal transduction times due to various anatomical and physiological characteristics such as body size and composition, sex, and age (Soudmand et al., 1982; Buschbacher, 1998; Fulöp et al., 2001) may influence the timing-dependent profiles of neural excitability and thus the effectiveness of Mstim-paired manual therapy. Anecdotally, physical therapists skilled in this clinical practice are able to adjust the stimulation intervals by observing responses in a patient based on their experience. However, this heuristic approach is unfortunately not fully generalizable. As a result, Mstim-paired rehabilitation has been difficult to standardize. Hence, it is imperative to develop an efficient method that helps determine the appropriate range of ISI for inducing neuromodulation in Mstim-paired manual therapy or Mstim-PAS for each individual. In addition, it is reported that this neuromodulation effectiveness is dependent on the particular phase of the sleep-wake/circadian cycle (Cohen et al., 2010; Lee et al., 2012).

### 1.2. Characterization of Mstim-Induced Neuromodulation

Potentiated synaptic excitability of M1 is assessed with the amplitude of motor evoked potential (MEP) via unpaired single-pulse TMS. Induced potentiation at this motor system level is analogous to the long-term potentiation (LTP) at the synaptic level, i.e., strengthened excitatory synapses due to repetitive timing-dependent excitation of presynaptic and postsynaptic neurons (Hebbian model) (Hebb, 1949; Bliss and Collingridge, 1993; Markram, 1997). At the synaptic level, LTP can be induced only when the postsynaptic neurons are excited immediately after the excitation of presynaptic neurons. Similarly, the largest and most consistent facilitation of M1 via neuromodulation is induced when cortical stimulation is applied 2–5 ms after the arrival of the Estim-induced sensory signal at the somatosensory cortex (Stefan, 2000; Stefan et al., 2002; Ziemann, 2004; Müller et al., 2007). With Estim, the arrival time can be determined for each individual with the first negative peak around 20 ms latency (N20) in somatosensory evoked potentials recorded through electroencephalography (EEG) (Wolters et al., 2003; Müller et al., 2007). The critical issue is that Estim-PAS can induce inhibition if the brain is stimulated 5–10 ms before the arrival of the sensory signal (Wolters et al., 2003; Ziemann, 2004; Müller et al., 2007). For example, Wolters et al. previously utilized the N20-P25 somatosensory evoked potential (SSEP) complex to evaluate the effect of PAS timing on neural plasticity (Wolters et al., 2005). The latency of the complex is important to clinical applications of PAS, as changes of even 15 ms in timing can reverse the direction of the neuromodulation (Wolters et al., 2005). Hence, the consequent neuromodulation with this type of paired stimulation can be inverted due to a subtle timing deviation.

Depending on stimulation intensity, Estim often causes a painful sensation. In addition, PAS with electrical stimulation requires tight synchronization between TMS and electrical nerve stimulation, needing skilled operators and long calibration time. In contrast, Mstim is more applicable and relevant to the actual clinical practice and desired muscle activity. Moreover, Mstim allows access to various muscles that cannot be individually accessed via electrical nerve stimulation.

As a simpler paradigm before this combined long-term effect is investigated, however, there are few studies that examined instantaneous and transient modulation of excitability of M1 due to Mstim. In Estim, excitability of M1 is inhibited at a latency of 19–50 ms, known as short-latency afferent inhibition (Tokimura et al., 2000), likely due to the direct thalamocortical projections to M1 via cholinergic paramedian thalamic nuclei (Lazzaro et al., 2000) and the projections from the primary sensory cortex to M1 (Ferezou et al., 2007; Aronoff et al., 2010), which recruit M1 interneurons that inhibit layer V pyramidal neurons (Lazzaro et al., 2000, 2007).

In contrast, Mstim of the periphery in the form of muscle stretch often produces transcortical long-latency stretch reflex with 50–150 ms latencies Rothwell et al. (1980); Shinohara et al. (2005), suggesting the induction of long-latency facilitation of M1. Impact-based Mstim to the muscle-tendon complex can stimulate various mechanoreceptors in the skin, tendon, muscle, and joint. These afferent inputs induced by Mstim can project to various supraspinal pathways other than those with Estim, potentially leading to not only net facilitation but also net inhibition of M1 in a latency-dependent manner.

**Figure 1:**
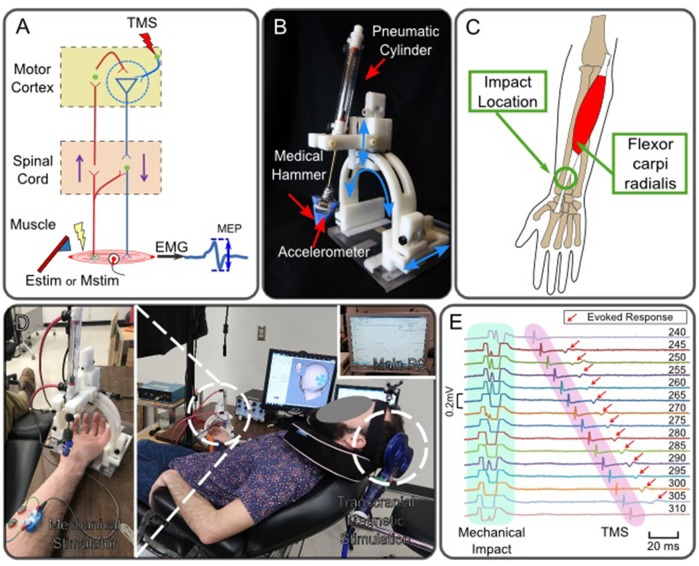
Robotic neuromodulation system paired with magnetic brain stimulation. **(A)** Schematic diagram of neuromodulation in the motor cortex induced by peripheral stimulation and observation of induced motor evoked potential. **(B)** Robotic tendon-tapping system. **(C)** Anatomical structure of Flexor carpi radialis muscle. The robotic tendon tapping device applies mechanical impact to the wrist tendon of the FCR muscle indicated by a circle. **(D)** Experimental setup of peripheral Mstim and measurement of neuromodulation by means of TMS; A subject is lying down on a bed experiencing Mstim and TMS. Both devices are operated by the main computer. **(E)** Enhanced MEP response with different intervals between Mstim and TMS (Subject 1). Values shown at the right indicate time intervals in milliseconds between t_valve_ and t_TMS_. t_Valve_ was fixed and t_TMS_ was changed. Artifacts were observed in EMG due to both Mstim and TMS. Artifacts due to Mstim are not full shown as the plots were trimmed to 0.2 mV and mechanical impact timings are not fully shown. Red arrows indicate MEP when Mstim and TMS are overlapped over the motor cortex. Note that no MEP was observed on the top and bottom traces where the timing interval between Mstim and TMS was outside of the effective ISI-W that observes enhanced MEP.

While long-term neuromodulation can be induced after a repetition of Estim-PAS or Mstim-paired manual therapy with an appropriate timing interval, a single application of peripheral stimulation can also induce transient neuromodulation in a time-dependent manner. The time-dependent profiles of neuromodulation in response to impact-based Mstim of muscles have been understudied because of the absence of a versatile and reliable mechanical stimulator that allows for timing adjustment. A combination of TMS and impact-based Mstim by using a high-timing-precision robotic tendon tapping device (Lacey et al., 2013, 2014; Ueda et al., 2014) was adopted to enable the exploration into Mstim-induced neuromodulation as shown in Figure 1A. In this study, MEPs were measured via electromyogram (EMG) of the FCR muscle to observe Mstim-induced neuromodulation as shown in Figure 1A. This configuration that pairs Mstim and TMS enables investigation of neuromodulation via different afferent nerve stimulation from one studied by using the conventional conditioned stimulation with Estim and TMS (Chen et al., 1999; Tokimura et al., 2000; Tamburin et al., 2005; Bikmullina et al., 2009; Devanne et al., 2009; Kojima et al., 2014). The form of mechanical tendon tapping will not induce joint motion unlike other work that utilized rapid joint extension as Mstim (Day et al., 1991).

### 1.3. Need for Individually Adjusting Mstim and TMS Timings

Due to the generally large variability in ISI windows (ISI-W) for observing Mstim-enhanced MEP in individuals, an adjustment procedure must be performed before actual neuroscientific research in every single subject. ISI-W can also vary depending on the experimental arrangement, including mechanical variability associated with the tested muscle and employed Mstim. Mstim is an indirect method to stimulate the peripheral sensory organs by applying physical perturbation in the form of transient changes in pressure, velocity and acceleration in the target peripheral tissues. These processes introduce additional dynamic factors, which lead to delayed and variable responses, such as skin and tendon stretch dynamics, muscle spindle discharge timings associated with the human sensorimotor system dynamics, in addition to air pressure propagation and development in the pneumatic system associated with the mechanical tendon tapping system.

The method of evaluating all responses across the set range and interval for identifying the ISI-W requires many data samples, which is laborious to the recipient of stimulation and makes the collection of a large data set time-consuming. For example, in the authors previous study (Kim et al., 2016), the initial range of ISIs for Mstim sub-threshold TMS neuromodulation was uniformly given to be 250 ms for all subjects where 12 Mstim trials were applied for 5 ms increments, totaling 600 Mstim trials. Including variable rest breaks between trials, average duration to complete data collection for each individual to determine the ISI-MEP profile was approximately 2 h (see section Experimental Procedure).

### 1.4. Our Approach

The goal of the current work is to develop statistical sampling and regression methods to efficiently model transient neuromodulations in the motor cortex via impact-based Mstim to muscles by means of a high-timing-precision robotic mechanical stimulator and its paired cortical stimulation. An experimental identification of the exact timing and magnitude of neuromodulation in M1 of an individual usually requires many test trials at different ISIs to individualize stimulation timings.

To mitigate this issue, the proposed procedure estimates individual ISI-W with sub-threshold TMS and Mstim. Statistical sampling and regression with relatively large time intervals (e.g., 5 ms) approximates the profile of Mstim-enhanced MEP with a reduced number of stimulation trials.

## 2. Materials and Methods

### 2.1. Design and Assessment of High-Timing Precision Tendon Tapping Robot

Msim-induced neuromodulation has not been well studied because of the absence of a basic mechanistic theory for this promising rehabilitation and a robust reliable system to implement interval-controlled Mstim. Compared with the conventional Estim, there is limited research on Mstim and its application to therapeutic exercises. To provide impact-based Mstim in a well-controlled manner, a pneumatically powered robotic tapper shown in Figure 1B (Lacey et al., 2013, 2014; Ueda et al., 2014) was utilized.

The developed robotic tendon tapping device enables paired Mstim of periphery and transcranical stimulation of the cortex (Lacey et al., 2014; Kim et al., 2016). This device is operated by a pneumatic linear cylinder (Airpel E9D20U, low-friction type, Airpot Corporation, Norwalk, CT) equipped with a medical hammer to apply impact-based mechanical stimulation to the target muscle in a form of tendon tapping as shown in Figure 1B. This pneumatic system creates tapping motion: the hammer linearly moves toward a target tendon, taps the tendon for 0.5 s, and retracts back to the original position. A pneumatic valve (MPYE-5-M5-010-B, Festo, Esslingen, Germany) is connected to the cylinder via a 7.5 m long pneumatic hose. A pressure sensor (SSI-P51-101, SSI Technologies, Inc., Janesville, WI, US) measures pressure in the upper chamber of the cylinder where constant pressure (60 psi) is applied from a pressure compressor. An accelerometer (MMA2202K, Freescale Semiconductor, Inc., Austin, TX, US) attached to the medical hammer detects the time when the hammer hits the tendon.

The tendon-tapping robot was designed to be adjustable to different locations and dimension of subjects by rotating and translating the hammer housing structure so that the hammer can tap the tendon from an appropriate angle. To allow for the future use of this device in functional magnetic resonance imaging (fMRI) environment to directly monitor the brain activity with robotic intervention, only MRI safe materials are used (Lacey et al., 2014) (Figure 1B). The only major ferromagnetic component is the FESTO pneumatic valve that is placed several meters away from the subject. This configuration helps to reduce electromagnetic interference on electromyographic and electroencephalographic measurements that would have had significant artifacts (Luck, 2014) if a non-shielded electromagnetic motor had been used to swing a hammer.

### 2.2. Experimental Procedure

Healthy adult subjects (*n* = 11) participated in this study from the pool of Georgia Institute of Technology, Atlanta Campus. All subjects were between 18 and 30 years and were right-handed without history of neurological disorders. The experimental procedure was approved by the Institutional Review Board (IRB) at Georgia Institute of Technology (Protocol number: H14191). Subjects were asked to read and sign a consent form before the experiment.

**Figure 2:**
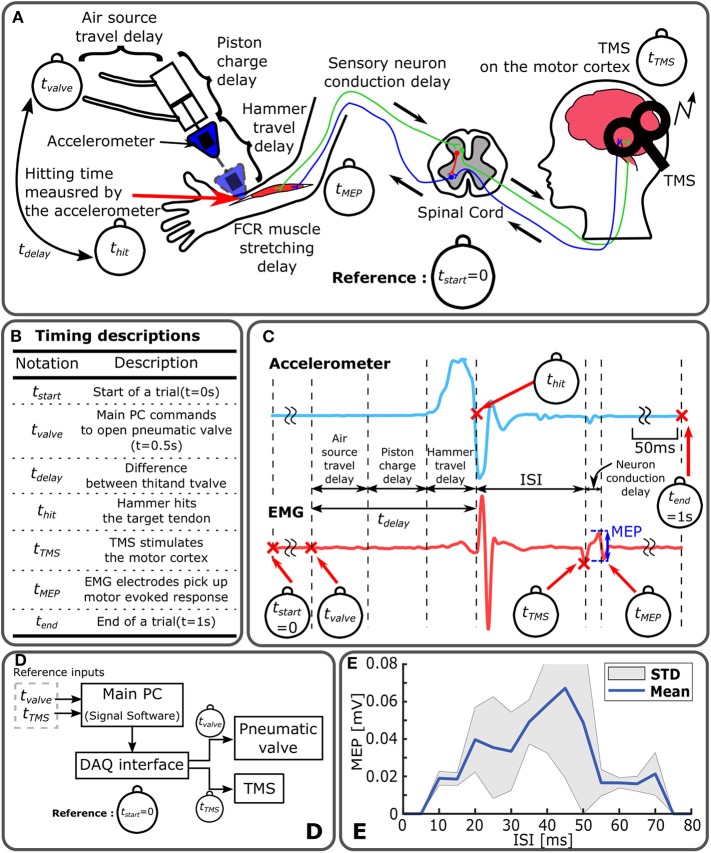
Timing control diagram of Mstim-induced neuromodulation and measurement of MEP. **(A)** Sources of time delay in Mstim-based robotic neuromodulation system. All event timings are based on t_Start_. **(B)** Timing descriptions. **(C)** Representative acceleration and EMG measurement. **(D)** System configuration diagram. Main PC operates Mstim and TMS devices based on t_valve_ and t_TMS_ via a DAQ interface. **(E)** Enhanced MEP ISI-W with Mstim and sub-threshold TMS. Enhanced MEP amplitude changes with varying ISI (t_TMS_-t_hit_). MEP was measured for ten times at every 5 ms increment of ISI. Effective ISI-w to observe enhanced MEP was 70 ms (from 5 to 75 ms) in this particular subject.

As shown in Figure 1D, a subject was supine on a bed experiencing Mstim from the robot as well as TMS. The timing of TMS and Mstim (i.e., *t*_TMS_ and *t*_valve_, respectively) were controlled by Signal (Cambridge Electronic Design Limited, UK). The tapping device applied Mstim to the wrist tendon connected to the flexor carpi radialis (FCR) muscle as shown in Figure 1C. The impact timing (i.e., *t*_hit_) was detected by the accelerometer attached on the hammer. The robotic system was configured as illustrated in Figure 2D. Two timing commands, *t*_valve_ and *t*_TMS_, were sent from the host PC. Resultant MEP amplitudes were recorded from a subject. To measure peak-to-peak MEP values of the FCR muscle for different ISIs, surface EMG was used. Two Ag-AgCl electrodes (E224N, In Vivo Metric, Healdsburg, CA, US) were placed on the FCR muscle belly as shown in Figure 1D.

An accelerometer mounted to the medical hammer was utilized to measure the acceleration of the hammer and detect the onset of hammer tendon impact (*t*_hit_). As shown in Figure 2C, *t*_hit_ was determined by detecting the time where the acceleration crossed zero for the first time after the initiation of hammer motion. Assuming negligible variability in *t*_TMS_, ISI was calculated by *ISI* = *t*_TMS_−*t*_hit_. Note that *t*_TMS_ and *t*_valve_ were reference commands in the system. *t*_hit_ is a resultant timing point which is *t*_hit_ = *t*_valve_+*t*_delay_ as shown in Figure 2B. In this paper, the acceleration was measured from five subjects out of eleven subjects.

**Figure 3:**
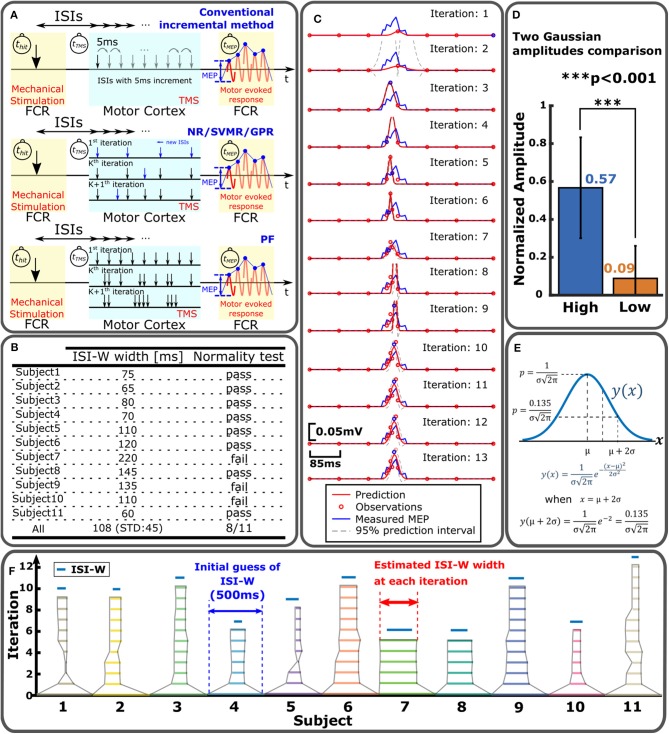
Sampling and statistical regression of enhanced MEP for individual ISI-W estimation. **(A)** MEP sampling procedures for different regression methods to estimate effective ISI-W. Top: conventional incremental measurement of MEP. Middle: NR/SVMR/GPR. Bottom: PF. **(B)** Normality test of enhanced MEP measurements. **(C)** Example estimation of enhanced MEP by NR. **(D)** Modeling of enhanced MEP profile with two Gaussian functions by NR. The amplitude of the first Gaussian function converged significantly greater than the amplitude of the second Gaussian; enhanced MEP profiles may be modeled by a single Gaussian function. **(E)** Determination of ISI-W from single-Gaussian fitting in NR and PF. Region within two standard deviations from the mean (i.e., two sigma) was used to determine ISI-W. **(F)** Result of ISI-W estimation with seven initial observations by GPR. Horizontal bar represents estimated ISI-W at each iteration. Blue bar on top is ISI-W determined from the conventional incremental measurement as ground-truth.

Enhanced MEP amplitudes were measured following the conventional procedure developed for Estim study. In the conventional method, the timing between *t*_TMS_ and *t*_valve_ was incrementally changed from 0 to 500 ms by 5 ms as shown in Figure 3A. Once all MEP amplitudes were recorded, the data was used for ground truth for MEP modeling and ISI-W estimation. A series of ISIs where MEP measurements were above the baseline (i.e., MEP with 100% resting motor threshold TMS) indicating enhancement was used to determine the effective ISI-W with sub-threshold TMS.

Note that in the conventional incremental procedure of MEP data collection, the experimenter in general expected a single distribution of MEP across ISI, and was allowed to manually terminate date collection when little or no MEP was observed after observing a distribution before reaching the longest end of searching time range of 500 ms. Without manual termination, a maximum of 600 trials was needed to incrementally sweep the entire search time range: Each mechanical stimulation took 1 s (1 s × 12 trials) followed by a 2 min rest. Among 12 MEP measurements at a single ISI, first two measurements were discarded and last 10 measurements were recorded. The first two measurements were discarded due to potential startle response. Two 5 min long breaks were given between sessions. As a result, typical time to collect data from one subject was: (1 s × 12 repetitions + 120 s rest) × 50 intervals + 600 s long rest × 2 = 130 min. By allowing the experimenter to decide and manually terminate, average number of trials was reduced to 260, which was still almost an hour.

### 2.3. Evaluation of Timing Precision of Tendon Tapping

Mechanical impact timing delay and precision of the tendon tapping robot were evaluated previously (Kim et al., 2016). Two types of timing evaluation were conducted by applying mechanical impact to (1) a force sensor (LCM703-50, Omega Engineering Inc., Stamford, CT) fixed to a rigid floor and (2) the forearm of human subjects (a total of four). In the first evaluation, the force sensor was used to detect the timing location of *t*_hit_. A total of 50 trials were performed. In the second measurement of impact application on human subjects, accelerometer readings were used to detect the hitting time (*t*_hit_) as illustrated in Figure 2C. Data was collected from average of 365 tapping trials per subject.

As expected, the tapping delay *t*_delay_ ranged from 172 to 188 ms for different subjects primarily due to slight differences in distance between the hammer's initial position and the target tendon position. However, within a single subject, highly repeatability was observed. By offsetting mean time delay between the subject, timing precision of mechanical impact application was determined to be 2 ms in STD across all the trials, and judged that the tapping precision was sufficiently high with small variability to meet the timing precision of Mstim.

### 2.4. Statistical Regression of Enhanced MEP and ISI-W Estimation

A total of four methods have been implemented and compared to each other: (1) Nonlinear Regression with Gaussian Model (NR), (2) Support Vector Machine Regression (SVMR), (3) Gaussian Process Regression (GPR), and (4) Particle Filter (PF). NR is a widely used regression method that assumes a parametric nonlinear model of experimental data including biological data (Motulsky and Christopoulos, 2010). Shapiro-Wilk Test was applied to the current MEP data. This test verifies the null hypothesis that the samples came from a normally distributed population. The mean MEP values at different ISIs were applied to this test to examine whether the MEP distribution is normal or not. Measured MEP distributions from eight subjects out of eleven did not reject the null hypothesis as shown in Figure 3B. Based on this normality test, a single-Gaussian Model was adopted as a parametric model to fit the measured MEP profiles for NR and PF. A two-Gaussian model was tested to see how two Gaussian peak amplitudes are determined in NR for all subjects (*n* = 11) for ten times each. In each trial, a greater amplitude was labeled as “High” and smaller amplitude was labeled as “Low” as shown in Figure 3D. Amplitudes were normalized for each subject for comparison across the subjects. Amplitude of the Gaussian with a higher amplitude was 6.3 times greater on average than the other Gaussian. In addition, in 70% of the trials, the amplitude of the second Gaussian converged to zero, supporting practical usefulness of single-Gaussian modeling while it would reduce precision to some extent. SVMR is a nonparametric regression method based on support vector machine (SVM). GPR is another nonparametric regression method that utilizes certain base functions that represent uncertainties in prediction as a Gaussian distribution, and may resemble the variability of multiple MEP measurements at a single ISI (Rasmussen and Williams, 2008). Nonparametric algorithms may be useful to model ISI-MEP data that does not necessarily fit a Gaussian model and perform regression with no assumption on the underlying function. On the other hand, nonparametric methods require many data points and relatively long processing time. PF was previously implemented by the authors (Takemura et al., 2018) where 30 particles were adopted.

The top row of Figure 3A shows the procedure of the incremental method that incrementally changes the interval between *t*_TMS_ and *t*_Valve_ by 5 ms to sweep through the predefined searching range. PF distributed 30 particles within the search range as shown in the third figure in Figure 3A. Each particle specified a certain ISI to observe MEP. Each particle's weight was updated based on its MEP measurement. Based on the locations and weights of particles, particles were redistributed in the next iteration to update the estimation of ISI-W. Detailed explanation on PF can be found in Takemura et al. (2018). NR, GPR, and SVMR first measured MEP for predetermined initial ISIs. ISI-W was estimated in an iterative fashion as shown in the second figure in Figure 3A. An acquisition function determined next ISI to observe MEP. This new observation updated the estimation of ISI-W. Detailed procedures of all four methods are given in Algorithms 1–4 in Supplementary Material. Figure 3C shows a representative estimation process with NR for one subject. The Gaussian model fits more closely to the measured MEP as regression progresses. In this particular trial, the initial number of observations was chosen to be 7 shown by red circles in the top plot of Figure 3C.

MEP amplitudes corresponding to initially chosen ISI values were recorded and stored in a form of an array. Arrays of ISI and MEP amplitudes were fed into the NR, SVMR, GPR, and PF algorithms. Next ISI to evaluate MEP was determined based on updated prediction, completing one iteration. New ISI and corresponding MEP were added to the arrays for the next iteration. This procedure continued until predefined stopping criteria were met that are given as follows: Variances at the lower and upper ends of the modeled distribution were evaluated. Iteration was terminated when variances in last five iterations of both the lower and upper ends were lower than 50, or iteration reached a maximum number of 99. The variance of 50 corresponds to 7 ms standard deviation of the last five lower and upper ends. This is slightly greater than the 5ms increment of ISI, which tolerates one interval long (5 ms) change of two ends location. The detailed procedures are explained in Algorithms 1–4 in Supplementary Material.

After the regression of MEP amplitude converged, a threshold method was applied to find two ends of the Gaussian model to determine its ISI-W. For a Gaussian distribution, the range within two standard deviations (i.e., two sigma, or 95%) was used as an estimation of effective ISI-W as shown in Figure 3E. 0.135 is the value of a normal distribution at two ends. For the nonparametric SVMR and GPR, the same amplitude of 0.135 was applied to determine the ISI-W for consistency. The final estimated profiles from these methods had hill-shaped curves similar to Gaussian curves (see Figure 3C and Figure S1). Figure 3F shows how estimation of ISI-W progresses with GPR.

Number of observations refers to the number of MEP measurements before the algorithm stops. Cross correlation between an estimated MEP curve and measured MEP profile as the ground-truth was evaluated. A correlation coefficient was determined using Pearson's linear correlation coefficient. F1 score was calculated by evaluating true positive, false positive and false negative. Using the two ends of both estimated ISI-W and measured ISI-W, F1 score produced a value ranging from 0 to 1 that represented the similarity between the estimated and measured ISI-W lengths. When the two time windows exactly matched, F1 score would become 1. Otherwise, F1 score would take a value <1. Percentage of convergence is the percentage of trials that have converged before reaching the maximum iterations of 99.

For these statistical regression estimations, MEPs of corresponding ISIs were measured and recorded in advance using the conventional incremental method. During the statistical regression estimation, the estimation algorithm retrieved necessary MEP measurements from the pool of data. Five subjects out of 11 had acceleration measurement of the hammer motion which could be used to narrow the search time range. Using this acceleration reading, ISI-W estimation results were analyzed in two different groups, one without acceleration measurements (for 11 subjects) and one with acceleration measurements (for five subjects) which collected 1,895 and 824 data points, respectively. For the group without the acceleration measurements, the search range was between *t*_Valve_ and the end of the trial. For the group with the acceleration measurement, *t*_hit_ detected by the accelerometer was used as the start of the search range.

## 3. Results

### 3.1. Assessment of High-Timing Precision Tendon Tapping Robot

To characterize timing-dependent transient neuromodulation due to Mstim, the mechanical tapping device must satisfy timing precision requirements. Compared with Estim, there are additional sources of time delay and variability in the developed pneumatic device, including: (1) air pressure propagation time in the pneumatic line, (2) pressure development time in the pneumatic chamber and pressure attenuation, (3) travel time of the hammer moving from its initial position to the target tendon position, and (4) non-linear mechanical interaction between the hammer material and skin/subcutaneous tissues. The sum of these factors produces a system delay between a pneumatic valve open command and resultant tendon tapping as illustrated in Figure 2A.

It should be noted that such delays can be compensated as long as the delay is repeatable. In other words, the speed of response of the mechanical tapping device is not important, but its timing precision is critical for the study of transient neuromodulation induced by Mstim. The developed device was designed to address this issue. A mean time delay was 195.5 ms with a standard deviation of 2 ms, which is an order of magnitude smaller than a typical ISI-W size due to Mstim (Kim et al., 2016), ensuring high repeatability of mechanical stimulation in time as shown in Figure S3. Furthermore, the timing of the onset of Mstim can be observed by using the accelerometer attached on the pneumatic hammer. Figure 2B shows all timing events. Figure 2C shows representative measurements of acceleration and EMG of the procedure. Note that for Mstim-induced neuromodulation, ISI is defined as the timing difference between t_Hit_ and t_TMS_.

### 3.2. Effective ISI for Observing Mstim-Enhanced MEP

Instantaneous and transient neuromodulation of M1 was explored by observing the emergence of the combined effects of sub-threshold Mstim to a wrist flexor tendon and sub-threshold TMS of M1 for a wrist flexor muscle (flexor carpi radialis) as shown in Figure 1C. Sub-threshold TMS (90% of resting motor threshold) was applied targeting the muscle at various ISI from Mstim, using the tendon tapping robot (Kim et al., 2016). See section Experimental Procedure for details. When sub-threshold Mstim or TMS alone was applied, the absence of an evoked response was confirmed as shown in Figure 1E top and bottom traces. With a combination of sub-threshold Mstim and TMS at various ISIs with 5 ms steps, evoked responses were observed (red arrows in the 2nd–14th traces) when the range of time interval from the Mstim robotic command to TMS was 60 ms (i.e., from 245 to 305 ms) in this setup. Within this 60 ms window, the combined effect of Mstim and sub-threshold TMS is substantial enough to activate the motor neurons to observe enhanced MEP. Since application of Mstim solely does not produce a long latency reflex response (Lacey et al., 2013), sub-threshold TMS was paired with Mstim to observe enhanced MEP due to Mstim in the muscle of interest Figure 2E shows a representative plot of enhanced MEP vs. ISI with sub-threshold TMS in one subject showing an effective ISI-W of 70 ms (subject 4). The measured effective ISI-W lengths of each individual are listed in Figure 3B. These lengths were determined based on MEPs measurement at ISIs using the conventional incremental method and individual ranges of ISI-W were variable as shown in the Figure 3B (mean 108 ms, std 45 ms). These measured effective ISI-W were used to compare and evaluate the performance of estimation methods (i.e., F1 score).

The length of effective ISI-W due to Mstim is comparable to our observation of the reflex-based latency window size in a hand muscle (50 ms) (Rothwell et al., 1980; Shinohara et al., 2005). When sub-threshold TMS was applied with sub-threshold Estim of the median nerve, however, the effective interval window size was 15–20 ms (Lacey et al., 2013) which is supported by previously reported work Poon et al. (2008). Hence, the effective ISI-W size with Mstim appears to be larger than Estim, (i.e., more dispersed response), possibly because of desynchronized activation of mechanoreceptors (e.g., muscle spindles) in response to Mstim. Mechanical peripheral stimulus produces a dispersed afferent volley in comparison to the impulse response to electrical stimulation. While this has been known since at least 1983 (Burke et al., 1983), limited work investigates the mechanism for this diffuse firing or its impact on stimulation based neuromodulation. These results confirm the presence of Mstim-induced transient neuromodulation of M1 and assures the importance of determining the effect in comparison to Estim.

### 3.3. Optimal Number of Initial Observations

NR, SVMR, and GPR procedures require initial data points (initial observation of MEP) to begin regression that must be specified by the user. The choice of the initial number of observations, or *n*_*ini*_, impacts the performance. The nominal searching period was given as [*t*_*valve*_ *t*_*valve*_+500 ms]. For example, evenly distributed initial ISIs within the searching period of 500 ms would be 500, 650, 850, 1,000 ms when *n*_*ini*_ = 4. With the use of the accelerometer, the range can be reduced to [*t*_*hit*_ *t*_*valve*_+500 ms], which may improve the performance.

**Figure 4:**
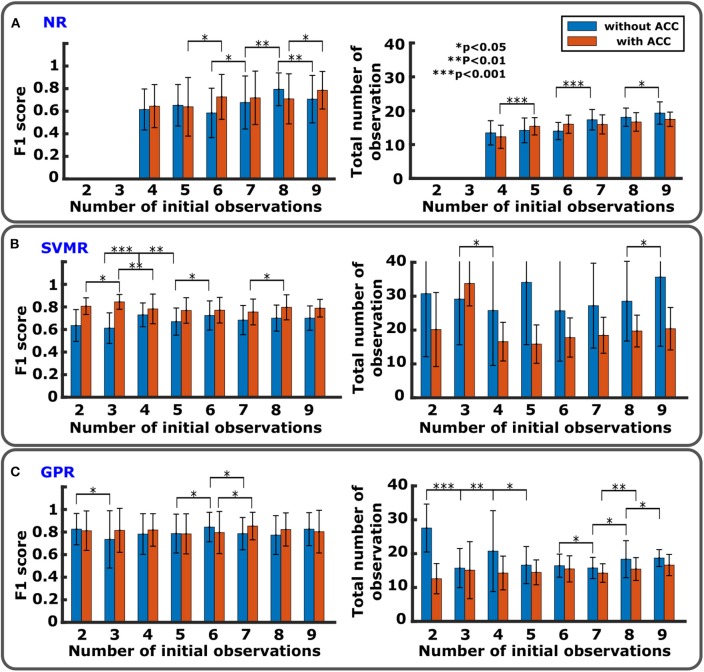
Comparison of regression performance with different numbers of initial observations. **(A)** NR with F1 score (left) and total number of observations (right). There is no data for *n*_*ini*_=2 and 3 since initial data points are too few to run the algorithm. **(B)** SVMR with F1 score (left) and total number of observations (right). **(C)** GPR with F1 score (left) and total number of observations (right).

**Figure 5:**
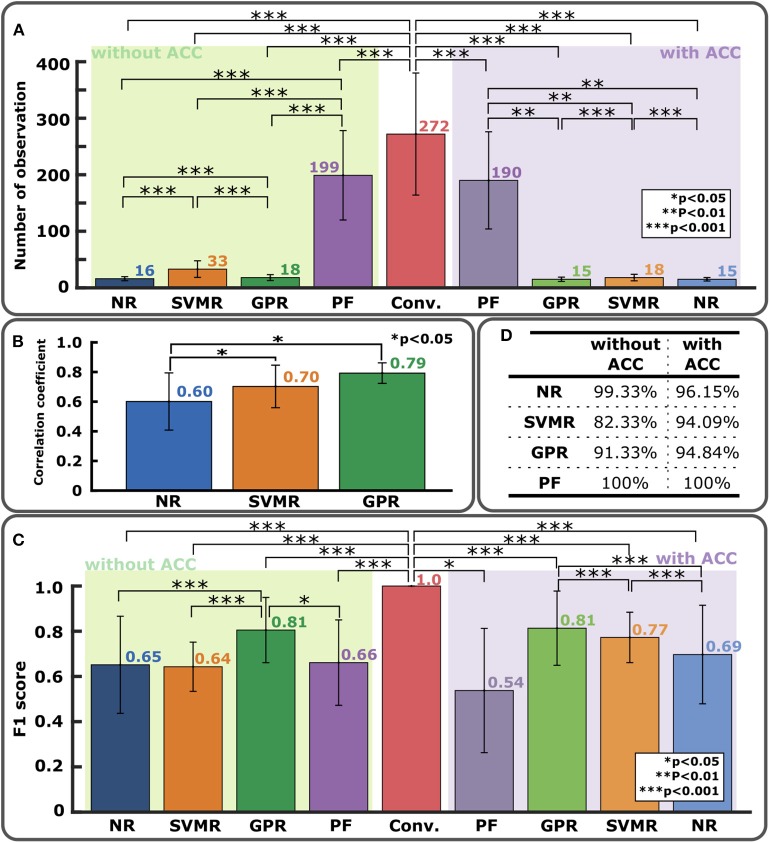
Comparison of performance on ISI-W estimation between different regression methods. **(A)** Numbers of total observations to obtain final estimation of ISI-W. For the conventional incremental measurement method (center bar), the total number of observations varied allowing experimenter's judgement to terminate the trial where a maximum of 600 observations were required to fully sweep the predetermined search window without manual termination. In the left half, bars show results without the use of the accelerometer (ACC) (*n* = 11). In the right half, bars show results with the use of the accelerometer (*n* = 5). **(B)** Correlation analysis of MEP profile estimation. Correlation coefficients comparing estimated MEP profiles and MEP measurements as ground-truth. Initial number of observations, 6, 7, and 7, were used for NR, SVMR, and GPR, respectively (*n* = 11). **(C)** Performance of ISI-W estimation evaluated by F1 scores. Data is displayed in the same manner as in **(A)**. Measurements from the conventional incremental method were used as ground-truth data shown in the center. **(D)** Percentage of convergence.

Figures 4A–C show comparisons of F1 score and number of total observations until convergence with *n*_*ini*_ from 2 to 9 with and without the accelerometer for NR, SVMR, and GPR. For the PF, except for the high rate of convergence as shown in Figure 5D, PF did not perform better than the other methods. In particular, PF still required a significantly greater number of trials. For this reason, the figure evaluated the initial number of observations only for NR, SVMR and GPR. For PF, 30 particles were used from our previous study (Takemura et al., 2018) that resulted in the best estimation performance among five different numbers of particles. In each graph, statistical significance was evaluated between neighboring bar plots within the same condition: without ACC and with ACC. For NR shown in Figure 4A, F1 score increased significantly from *n*_*ini*_=7 to *n*_*ini*_=8 without ACC and increased from *n*_*ini*_ = 6 to *n*_*ini*_ = 9 with ACC. Total number of observations was low for *n*_*ini*_ < 7 without ACC and *n*_*ini*_ < 5 with ACC. For SVMR shown in Figure 4B, F1 score took the highest value for *n*_*ini*_ = 4 without ACC and for *n*_*ini*_ = 3 with ACC. Total number of observations was low between *n*_*ini*_ = 4 and *n*_*ini*_ = 8 without ACC and low after *n*_*ini*_ > 3 for with ACC. For GPR shown in Figure 4C, F1 score took the highest value for *n*_*ini*_=6 without ACC and *n*_*ini*_ <8 with ACC. Total number of observations was low for *n*_*ini*_ >2 with ACC and low for *n*_*ini*_ <8 with ACC.

Practically at least 1–2 non-zero enhanced MEP responses must be observed as initial data points for a regression algorithm to successfully progress. Given average ISI-W of 108 ms in this dataset, *n*_*ini*_= 6 or 7 would meet this requirement.

### 3.4. Reduction of Mstim-TMS Trials for Individual ISI Window Estimation

Previous studies utilized a fixed ISI or a few manually-chosen ISIs (Stefan, 2000; Kumpulainen et al., 2012) to characterize neural responses associated with Estim. It should be noted that these ISIs were mostly determined based on empirical knowledge of neural conduction times. Other previous studies (Wolters et al., 2003, 2005) incrementally varied the interval between TMS and Estim to identify an ISI which would achieve the highest long-term potentiation in MEP amplitude. The presented work applied statistical sampling and regression methods to reduce trials for the estimation of individual ISI-MEP profiles by applying systematically chosen ISIs between Mstim and TMS, instead of incrementally sweeping a predetermined range of ISI.

In addition to individual differences, usually large variability in MEPs is observed in a single subject. ISI-W estimation methods must consider the stochastic nature of the human neuromotor system responses as shown in Figure 2E where large variation in MEP amplitudes was observed. Particle filtering technique was applied earlier (Takemura et al., 2018) in which particles determined when to apply TMS relative to Mstim to update an estimate of the MEP profile in an iterative manner. ISI-W estimation based on particle filtering showed potential to reduce the required number of observations. Other statistical regression methods were applied for further improvement of ISI-W estimation performance as shown in Figure 3A. In particular, a performance comparison was conducted between four statistical estimation methods: (1) Nonlinear Regression with Gaussian Model (NR), (2) Support Vector Machine Regression (SVMR), and (3) Gaussian Process (GP), in addition to previously reported, (4) Particle Filter (PF). Details are described in section Materials and Methods.

All of the regression methods required a certain number of initial observations distributed within an initial guess of ISI-W. When the accelerometer is available, it can detect the onset of Mstim or *t*_*hit*_. The period between *t*_*valve*_ and *t*_*hit*_ can be excluded from regression as facilitation must occur after *t*_*hit*_. The use of the accelerometer helps to narrow the searching range that may improve performance.

Performance was evaluated based on (1) total number of observations, (2) cross correlation, (3) F1 score, and (4) percentage of convergence. Four regression methods and the conventional method were compared using a significance level (alpha) of 0.05 with the null hypothesis that there is no significant difference between methods. Effect sizes are listed in the Supplementary Material.

Number of observations required for convergence is shown in Figure 5A. All the regression methods significantly reduced the number of observations compared with the conventional incremental method that required an average of 272 observations when the experimenter was allowed to manually terminate data collection. While PF improved the performance, PF still required many more observations and produced greater variance than NR, SVMR, and GPR did. On average, NR, SVMR, and GPR required an order of magnitude smaller observations than PF and the conventional incremental method with smaller variance. The use of the accelerometer further improved the performance by reducing number of observations up to 56%. The result of cross correlation analysis is shown in Figure 5B evaluating how much estimated MEP profiles were close to the profiles from full measurements as the ground truth. GPR's correlation coefficient was 31.67% higher than that of NR (*p* < 0.05), and SVMR's correlation coefficient was 16.67% higher than that of NR (*p* < 0.05). There was no statistical significance observed between SVMR and GPR. F1 score would be an appropriate evaluation metric to evaluate the accuracy of ISI-W estimation as shown in Figure 5C. GPR obtained F1 score greater than 0.8. The use of the accelerometer overall improved the performance. As shown in Figure 5D, only a small number of cases did not converge and reached the maximum number of iterations, showing satisfactory robustness to different data sets.

## 4. Discussion

Individual differences in physiological characteristics (See Paired Associative Stimulation for Neural Facilitation section) may result in variability in effective ISI-W in individuals. With sub-threshold TMS and Mstim, a single distribution of MEP across ISI is expected. Parametric statistical regression methods were applied to find effective ISI-w with the expectation of single distribution. The estimation results were close to the measured ISI-W, capturing individual variability.

Due to the generally large variability in effective ISI-W in individuals, an adjustment procedure must be performed before actual neuroscientific research in every single subject. The method of evaluating ISI requires many data samples and times. However, to the best of the author's knowledge, there is no study reporting determination of effective ISIs efficiently for individuals. These statistical regression methods would provide ways to determine effective ISIs for each individual by monitoring the subjects' MEP with reduced number of stimulation trials.

### 4.1. Gaussian Modeling of Mstim-Induced MEP Enhancement

For Estim-induced neuromodulation in terms of conditioned stimulation, other groups reported correlations between ISI and MEP amplitudes (Chen et al., 1999; Tokimura et al., 2000; Sailer, 2003; Bikmullina et al., 2009; Kojima et al., 2014). Mathematical representation of MEP induced by peripheral or cortical stimulation has been reported in the literature. For example, a statistical model of MEP induced by electrical pulse trains to simulate the firing pattern of MEPs was proposed (Ma et al., 2011). Another work developed a regression MEP model with different TMS intensities (i.e., input-output curve) (Goetz and Peterchev, 2012; Goetz et al., 2014). Gaussian modeling was adopted to fit the ISI-MEP relationship with a different number of Gaussian functions (Delvendahl et al., 2012; Cirillo and Perez, 2015; Cirillo et al., 2015; Kallioniemi et al., 2018; Mohammadi et al., 2018).

To the best of our knowledge, there are even fewer works that studied ISI-MEP association with Mstim and its modeling by using an automated tendon-tapping robot. The majority of MEP profiles associated with ISI reported in the literature exhibited a single MEP distribution as well as the ones collected in this paper (see Supplementary Material for raw MEP data) along ISI as shown in Figure 2E. While the objectives of Gaussian curve-fitting in the past studies are different from our objective of time-efficient ISI-W estimation, the use of Gaussian models seems a reasonable choice. As far as Mstim with sub-threshold TMS for effective ISI-W estimation is concerned, a single-Gaussian model may be sufficient for parametric modeling of enhanced MEP profiles.

With an oversimplified single Gaussian model, the speed of convergence would be improved and issues associated with over-fitting would be resolved. This simplified methods, PF and NR, were suitable for robust estimation of individual ISI-W as shown in Figure 5D. On the other hand, nonparametric methods, SVMR and GPR, were applied, considering variability in MEP profiles. They resulted in ISI-W estimation with better precision.

### 4.2. Limitation of This Study

In this study, effective ISI-Ws were estimated for each individual using four different methods and the results were evaluated. Since this study evaluated the overall estimation performance across all subjects, potential between-subject variability may not have been fully accounted for. Four estimation methods could be further optimized by tuning parameters to improve estimation performance.

## 5. Conclusion

In this work, a robotically enabled experimental procedure was applied for the study of neuromodulation induced by peripheral mechanical stimulation. This estimation procedure was enabled by high timing precision of the tendon tapping robot (STD < 5 ms). The size of ISI-W that observed enhanced MEP with Mstim by means of tendon tapping was found to be larger than that with sub-threshold Estim of the median nerve, possibly due to different involvement of mechanoreceptors. A single-Gaussian model was applied to enhanced MEP profiles for parametric regression. The combination of robotic tendon tapping and statistical regression can reduce the number of observations to individually determine effective ISI to observe enhanced MEP up to 6.5 % (NR with ACC), leading to reduction of physical burden on the subject who would otherwise receive many stimulation trials with high intensity for more than 2 h. Parametric models (NR and PF) that utilized a single-Gaussian model achieved high convergence. Regarding which estimation method should be used, the presented comparison indicated that the considered estimation methods performed differently in terms of various evaluation metrics. While there was no single method that outperformed the other methods, GPR would be a reasonable choice to achieve an ISI-W length close to the measured ISI-W length, resulting in a high F1 score. If the priority is the reduction of the total number of stimulation trials while achieving a high rate of convergence, NR should be chosen. Individual estimation methods could be further optimized by tuning parameters.

This line of research is expected to produce a reliable tool, which will clarify unique neuromodulations of the motor cortex with an application of robotic Mstim to muscles, in comparison to the conventional Estim of a peripheral nerve, and its paired central stimulation. The paired stimulation technique may be extended to study not only excitatory neuromodulation, but also inhibitory neuromodulation, when applied with supra-threshold TMS. This technique may also be used to evaluate neural plasticity with Mstim-TMS paired stimulation. Statistical regression methods would be able to capture individual effective ISI-W that is expected to have larger variability in individuals with neurological disorders compared with healthy individuals. The outcomes might allow for evidence-based implementation of mechanical stimulation paired with brain stimulation into individually tailored robotic rehabilitation for hemiparetic stroke survivors and possibly other disabilities.

## Data Availability Statement

All datasets generated for this study are included in the article/Supplementary Material.

## Ethics Statement

The studies involving human participants were reviewed and approved by Kelly A. Winn Georgia Tech's Central Institutional Review Board (IRB). The patients/participants provided their written informed consent to participate in this study.

## Author Contributions

MS and JU conceived and supervised the research. EK and JU conceptualized the ideal of applying statistical sampling and regression to the characterization of transient neuromodulation with paired mechanical and sub-threshold brain stimulation. EK and WM designed the studies, implemented the hardware and software, performed the experiments, and analyzed the data. EK and JU drafted the manuscript with contributions from WM and MS.

### Conflict of Interest

The authors declare that the research was conducted in the absence of any commercial or financial relationships that could be construed as a potential conflict of interest.

## Supplementary Material

Table S1 Measured MEP data of eleven subjects.

Table S2 MEP regression results.

Video 1 Robotic tapping device introduction and experimental procedure.
