# Supplementary material for: Statistical Inter-stimulus Interval Window Estimation for Transient Neuromodulation via Paired Mechanical and Brain Stimulation
Source: Front Neurorobot. 2020 Feb 3;14:1. doi: 10.3389/fnbot.2020.00001 (PMC7010981; doi:10.3389/fnbot.2020.00001)
Supplement: Supplementary file 4 [file Data_Sheet_1.PDF]

frontiers

Supplementary Material

1 REGRESSION ALGORITHMS

NR, SVMR, GPR and PF algorithms used for MEP modeling are shown in Algorithms 1,2,3 and 4.

Algorithm 1 Nonlinear Regression for estimating individual ISI-W

```
procedure NONLINEAR REGRESSION
    {t_TMSinitial}                                              ▷ Determine initial t_TMS
    iteration ← 1
    t_TMS = t_TMSinitial                                        ▷ Update t_TMS
    while variance1 ≥ 50 and variance2 ≥ 50 and iteration ≤ 100 do     ▷ Stopping criteria
        get {MEP(t_TMS)}                                        ▷ Measure MEP for each corresponding t_TMS
        y_regression = f(t_TMS, MEP)                            ▷ Regression result from NR
        y_regression(ISI − W_start) = max(y_regression) ∗ 0.135 ▷ Find ISI − W_start
        y_regression(ISI − W_end) = max(y_regression) ∗ 0.135   ▷ Find ISI − W_end
        t_TMSnext ← acquisitionfunction                         ▷ Determine next t_TMS from acquisition function
        t_TMS = t_TMSnext                                       ▷ Update t_TMS
        if length(ISI − W_start ≥ 5) then
            variance1 = variance(ISI − W_start(end − 5 : end))  ▷ Check variance
            variance2 = variance(ISI − W_end(end − 5 : end))    ▷ Check variance
        end if
        iteration ← iteration + 1                               ▷ Repeat until convergence
    end while
return y_regression
end procedure
```

Algorithm 2 Support Vector Machine Regression for estimating individual ISI-W

```
procedure SUPPORT VECTOR MACHINE REGRESSION
    {t_TMSinitial}                                              ▷ Determine initial t_TMS
    iteration ← 1
    t_TMS = t_TMSinitial                                        ▷ Update t_TMS
    while variance1 ≥ 50 and variance2 ≥ 50 and iteration ≤ 100 do     ▷ Stopping criteria
        get {MEP(t_TMS)}                                        ▷ Measure MEP for each corresponding t_TMS
        y_regression = f(t_TMS, MEP)                            ▷ Regression result from SVMR
        y_regression(ISI − W_start) = max(y_regression) ∗ 0.135 ▷ Find ISI − W_start
        y_regression(ISI − W_end) = max(y_regression) ∗ 0.135   ▷ Find ISI − W_end
        t_TMSnext ← acquisitionfunction                         ▷ Determine next t_TMS from acquisition function
        t_TMS = t_TMSnext                                       ▷ Update t_TMS
        if length(ISI − W_start ≥ 5) then
            variance1 = variance(ISI − W_start(end − 5 : end))  ▷ Check variance
            variance2 = variance(ISI − W_end(end − 5 : end))    ▷ Check variance
        end if
        iteration ← iteration + 1                               ▷ Repeat until convergence
    end while
return y_regression
end procedure
```

Algorithm 3 Gaussian Process Regression for estimating individual ISI-W

procedure GAUSSIAN PROCESS REGRESSION
  $\{t_{TMSinitial}\}$ ▷ Determine initial t_{TMS}
  $iteration \leftarrow 1$
  $t_{TMS} = t_{TMSinitial}$ ▷ Update t_{TMS}
  while $variance1 \geq 50$ and $variance2 \geq 50$ and $iteration \leq 100$ **do** ▷ Stopping criteria
    get $\{MEP(t_{TMS})\}$ ▷ Measure MEP for each corresponding t_{TMS}
    $y_{regression} = f(t_{TMS}, MEP)$ ▷ Regression result from GPR
    $y_{regression}(ISI - W_{start}) = max(y_{regression}) * 0.135$ ▷ Find $ISI - W_{start}$
    $y_{regression}(ISI - W_{end}) = max(y_{regression}) * 0.135$ ▷ Find $ISI - W_{end}$
    $t_{TMSnext} \leftarrow acquisitionfunction$ ▷ Determine next t_{TMS} from acquisition function
    $t_{TMS} = t_{TMSnext}$ ▷ Update t_{TMS}
    if length($ISI - W_{start} \geq 5$) **then**
      $variance1 = variance(ISI - W_{start}(end - 5 : end))$ ▷ Check variance
      $variance2 = variance(ISI - W_{end}(end - 5 : end))$ ▷ Check variance
    end if
    $iteration \leftarrow iteration + 1$ ▷ Repeat until convergence
  end while
return $y_{regression}$
end procedure

Algorithm 4 Particle filter for estimating individual ISI-W

procedure PARTICLEFILTER
  $\{t_0^{(1:M)}, \omega_0^{(1:M)}\} \sim p_0(.)$ ▷ Initialize particles with discrete times (t_0) and weights(ω_0)
  $k \leftarrow 1$ ▷ iteration
  while $variance \geq \epsilon^2$ **do**
    for $i \leftarrow 1, M$ **do** ▷ M is the number of particles
      sample $t_k^{(i)} \sim p\left(t_k | v_k, t_{k-1}^{(i)}\right)$ ▷ Redistribute particles. v_k represents the human system noise
      $\omega_k^{(i)} = p\left(z_k | t_k^{(i)}\right)$ ▷ Update weights based on MEP observation
    end for
    $\omega_k^{(i)} = \omega_k^{(i)} / \sum_{i=1}^{M} \omega_k^{(i)}$ ▷ Normalization
    $\{t_k^{(1:M)}, \omega_k^{(i)}\}$ =Resample$(t_k^{(1:M)}, \omega_k^{(1:M)})$
    $p(t_k|z_k) = \sum_{i=1}^{M} \omega_k^{(i)} \delta(t_k - t_k^{(i)})$
    $E\left[p(t_k|z_k)\right] = \sum_{i=1}^{M} \omega_k^{(i)} t_k^{(i)}$ ▷ Posterior expectation
    $k \leftarrow k + 1$ ▷ Repeat until convergence
  end while
return $E\left[p(t_k|z_k)\right], p(t_k|z_k)$
end procedure

2 MEP MEASUREMENTS AND REGRESSION RESULTS

Fig. S1 shows the measurement of enhanced MEP amplitudes that are used as the ground truth. Fig. S2 shows results of NR, SVMR, GRP and PF modeling MEP profiles. For these particular estimated MEP profiles, the estimations were done without using accelerometer reading and started with n_{ini}=7. Among the methods, GPR tends to closely reproduce MEP profile of the ground truth. This is observed in the highest F1-score of the GPR. This is probably because of the characteristic of GPR where the covariance is almost unity when input observations, or ISIs, are close in distance, or zero when they are highly distinct. This characteristic achieves MEP amplitude estimation near observed points of MEP. Due to nonparametric regression, SVMR tends to reproduce the overall shape of MEP amplitude including artifact as shown in Figure S2. This characteristic increases the number of iteration in SVMR algorithm to meet the stopping criteria. NR and PF used a single-Gaussian model that demonstrated robustness to measurement artifacts in Figure S1.

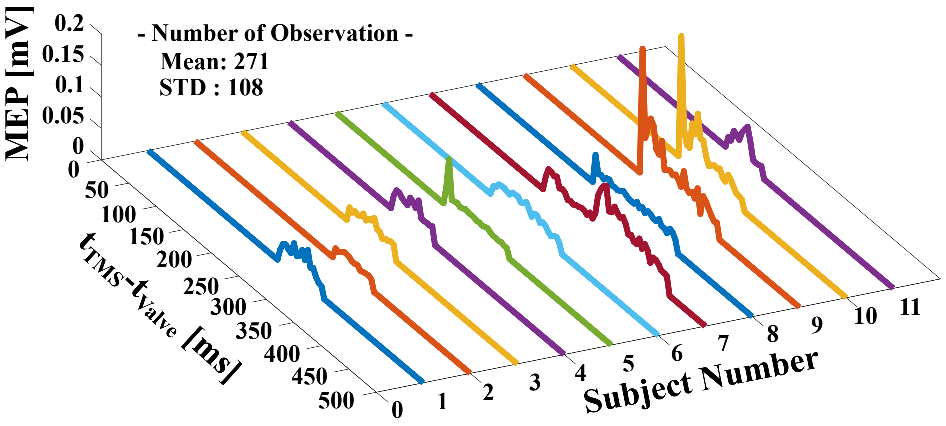

Figure S1: MEP Measurement of Mstim with sub-threshold TMS (Ground Truth). Note that for subjects 5,8, 9 and 10, mechanical artifacts due to skin stretch or spinal reflex were partially observed at the beginning of EMG measurements.

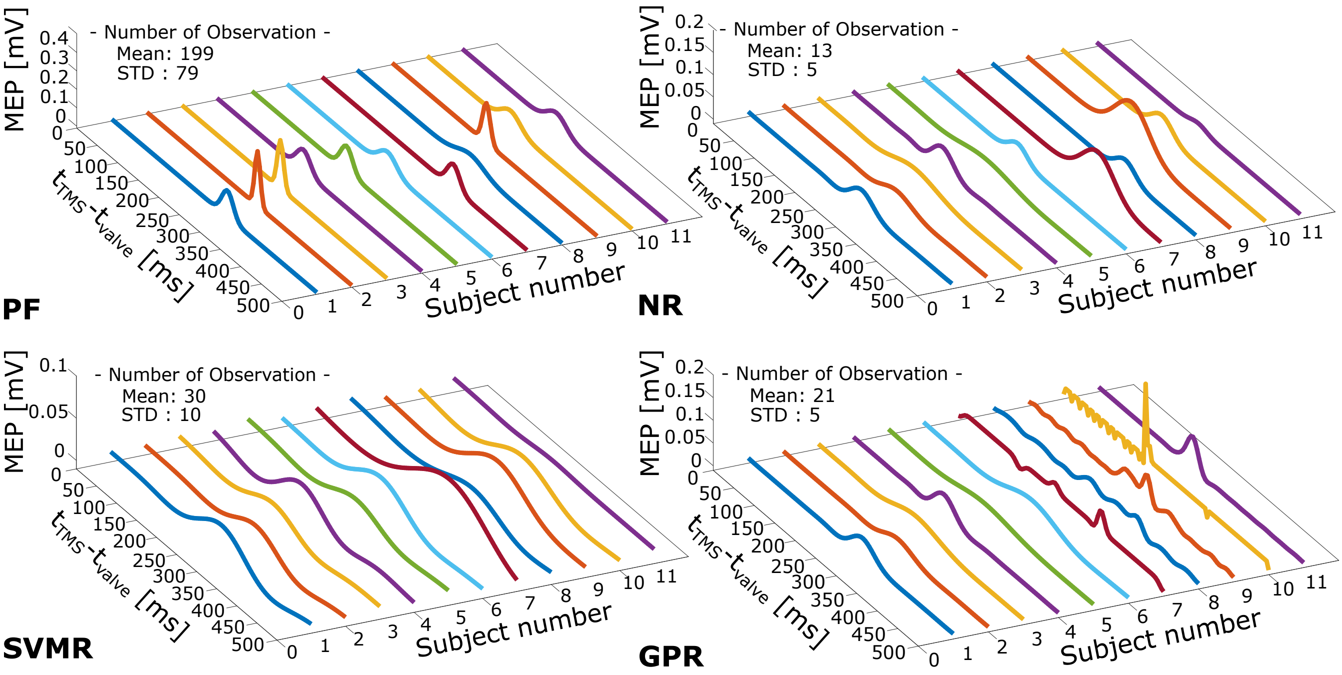

Figure S2: Regression results (PF, NR, SVMR, and GRP)

2.1 Mstim timing precision analysis

Fig. S3 shows the result of timing precision analysis of t_{hit} for five subjects. Mean t_{hit} values between the subjects were different with statistical significance, supporting the need for individual timing adjustment. Although individual subject's arm physical characteristics may have contributed to the variability in t_{delay}, standard deviation of impact timing in each subject was found to be less than 2ms, which demonstrates high-timing precision of the tapping device.

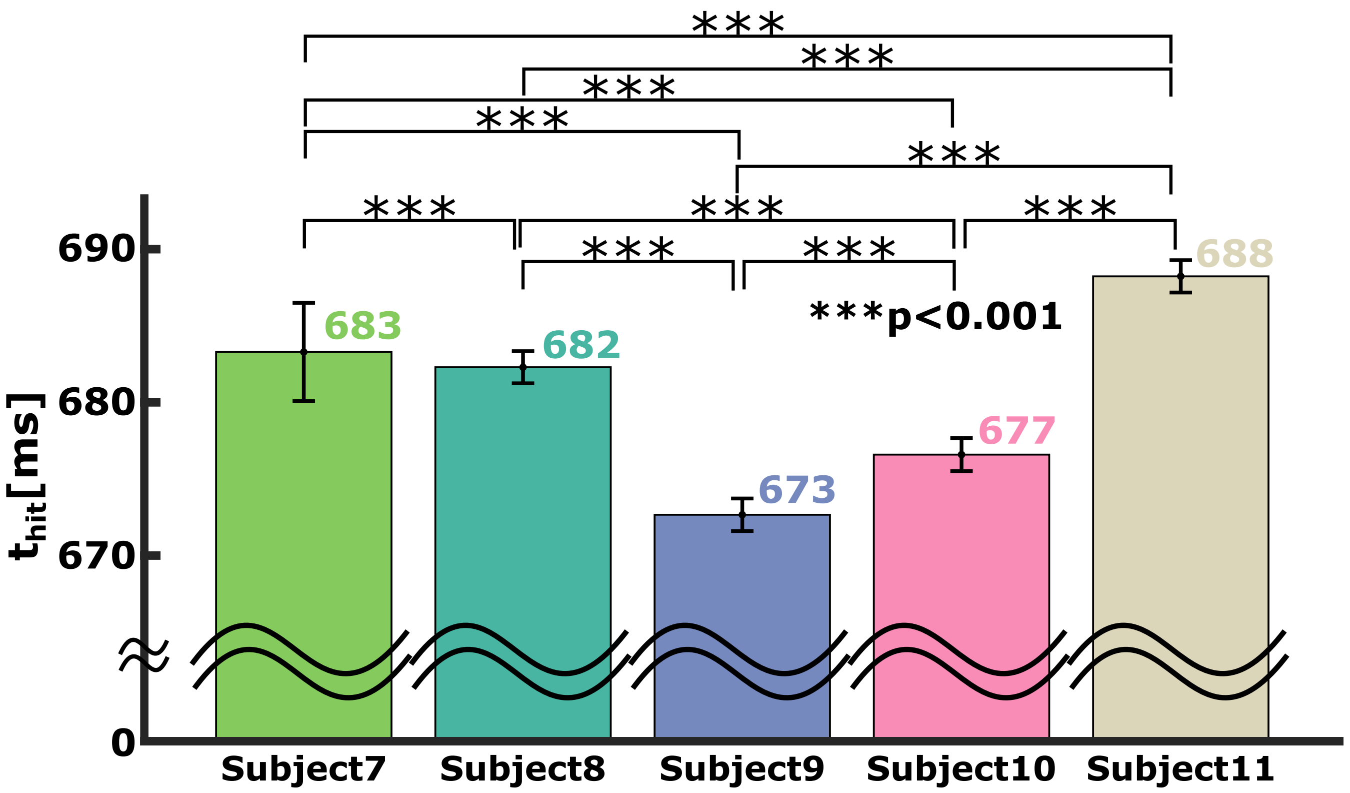

Figure S3: t_{hit} timing precision analysis (n=5)

2.2 Effect sizes between groups

Four regression methods and the conventional method were compared using a significance level (alpha) of 0.05 with the null hypothesis that there is no significant difference between methods. Effect sizes in terms of total number of observations and F1 score are shown in table S1.

Table S1. Effect sizes between estimation methods

	Number of Observations (without ACC)	Number of Observations (with ACC)	F1 score (without ACC)	F1 score (with ACC)
**NR-GPR**	0.43	0.07	0.77	0.58
**NR-SVMR**	1.24	0.56	0.05	0.42
**NR-PF**	7.03	6.98	0.04	0.72
**GPR-SVMR**	1.12	0.58	1.07	0.29
**GPR-PF**	6.89	6.68	0.98	1.61
**SVMR-PF**	5.91	6.26	0.16	1.93
**NR-CONV**	7.09	4.95	3	3.14
**GPR-CONV**	6.99	4.76	5.54	4.93
**SVMR-CONV**	6.41	4.51	5.5	6.52
**PF-CONV**	0.71	0.75	3.04	2.04
